# Clustering functional data using forward search based on functional spatial ranks with medical applications

**DOI:** 10.1177/09622802211002865

**Published:** 2021-11-10

**Authors:** Mohammed Baragilly, Hend Gabr, Brian H Willis

**Affiliations:** 1 Department of Mathematics, Insurance and Applied Statistics, Helwan University, Helwan, Egypt; 2 Institute of Applied Health Research, University of Birmingham, Birmingham, UK; 3 Faculty of Commerce, Menoufia University, Al Minufya, Egypt

**Keywords:** Cluster analysis, forward search, functional data, nonparametric methods, spatial ranks

## Abstract

Cluster analysis of functional data is finding increasing application in the field of medical research and statistics. Here we introduce a functional version of the forward search methodology for the purpose of functional data clustering. The proposed forward search algorithm is based on the functional spatial ranks and is a data-driven non-parametric method. It does not require any preprocessing functional data steps, nor does it require any dimension reduction before clustering. The Forward Search Based on Functional Spatial Rank (FSFSR) algorithm identifies the number of clusters in the curves and provides the basis for the accurate assignment of each curve to its cluster. We apply it to three simulated datasets and two real medical datasets, and compare it with six other standard methods. Based on both simulated and real data, the FSFSR algorithm identifies the correct number of clusters. Furthermore, when compared with six standard methods used for clustering and classification, it records the lowest misclassification rate. We conclude that the FSFSR algorithm has the potential to cluster and classify functional data.

## 1 Introduction

In many medical applications, the observed data may be assumed to have arisen from a continuous curve or higher dimensional surface that is described by some function. Thus, the glucose levels as measured by a continuous glucose monitor or the tracings generated by an electroencephalogram of a patient are both examples where a continuous function may be used to help describe the underlying data.

In both of these examples, a single function is assumed to underlie the data for an individual patient so that for a sample of patients there is a sample of functions in which the data are observed at particular points. In this regard, the function is the element of interest^1^ and analysis of the shape of these functions can help inform decisions on classification and prediction.^2,3^

Functional data analysis describes the statistical methods and techniques that are used to explore functional data.^1^ The random variable is a functional, that is, a space of functions, defined on some continuous interval such as time.^1,2^ Thus, each realization of the variable is a function providing infinitely dimensional data, and the space of functions is generally assumed to be a Hilbert space.^4^ Although our concern here will be with univariate functional data, sometimes multivariate functional data may be of interest.^1,5,6^ In practice, whichever the type of data, the functions are often sampled at a finite set of points.

In many situations, we need to know the hidden structure that explains how these curves and functions vary from one group to another. Thus, in the study of childhood obesity, growth curves of body measurements may be used to group children using a cluster method.^7^ One such cluster method, *k*-means clustering, has been used to identify patterns of multi-morbidity and check whether these patterns are stable over time for a cohort of older people.^8^

For many of the cluster methods used on functional data, the number of clusters is assumed to be fixed a priori. This makes determining the optimal number of clusters in the functional data important and motivates this research. Thus, the method proposed here can be used to identify the number of clusters and is a development on the forward search originally used to identify outliers in multivariate data^9,10^ and later, as a clustering method.^11^

Here we use a forward search based on functional spatial ranks to analyze functional data. This extends previous work that introduced the forward search based on spatial ranks for the cluster analysis of multivariate data.^12^ The functional forward search introduced here is based on the random start forward search,^13^ and can be considered a new raw-data method that obviates the need for dimension reduction, since it performs the clustering directly on the discrete observation of the curves or functions.

It is a non-parametric method that can be used to determine the number of clusters, and assign each curve to its cluster. When compared with existing methods using different numerical examples from real data, it is shown to be an effective tool in clustering analysis.

The paper is organized as follows. In section 2, we discuss the curse of dimensionality in the traditional random start forward search method and the potential of using the forward search based on functional spatial ranks. In section 3, we propose the functional forward search algorithm based on functional spatial ranks. In section 4, we compare the proposed method with other functional data clustering methods using numerical examples before ending with the discussion in section 5.

## 2 The curse of dimensionality in the traditional forward search

The term “curse of dimensionality” was introduced by Bellman.^14^ It refers to all problems caused by the analysis of high-dimensional data and, in general, arises from a relative sparsity of observations. For example, in order to run the traditional forward search algorithm based on Mahalanobis distances, we need to choose an initial subset *S(m)*, with *m = d + 1* for some dimension, *d*. Practically, this is not difficult if the number of available observations is large compared to the number of variables, although the traditional forward search algorithm becomes less efficient as the dimension *d* grows.

In contrast, when the number of observations is small compared to the number of variables it is not possible to estimate the variance–covariance matrix so the algorithm cannot proceed. Strictly, the traditional forward search based on Mahalanobis distances^11^ cannot be applied to functional data owing to the random variables taking values into an infinite dimensional space. However, in practice the data consist of curves that have been sampled at a finite set of points, hence it is still possible to use forward search methods providing the dimension, *d* is less than the underlying sample size.

The forward search, like all multivariate methods based on Mahalanobis distances, suffers when the dimension grows. Since it starts with subsets of size *d + 1*, it is unable to identify clusters of a size less than *d*. Thus, when both the number of variables is very big and some of the clusters are of a small size, this algorithm will lead to information loss about the number of clusters to be determined.

In contrast, using a forward search based on spatial ranks for clustering multivariate data overcomes this issue,^12^ as it can be started with subsets of any size since the rank of any observation $x\in \;R^{d}$ with respect to a single data point is always 1.^12^ Similarly, we can use the non-parametric forward search based on the functional spatial ranks for clustering functional data, even though functional data is intrinsically different from multivariate data. This algorithm is described in more detail in the next section.

## 3 The functional forward search based on functional spatial ranks

In this section, we propose a functional forward search algorithm based on functional spatial ranks. Part of the novelty of this algorithm is that unlike the traditional forward search algorithm, it works with functional data. Furthermore, as a raw data method it determines the number of clusters from the data without any need for parameter estimation. A key element is the need to extend $\mathrm{sign}\left(x\right)$ and $\mathrm{Rank}\left(x\right)$ naturally from $R^{d}$ to any infinite-dimensional Hilbert space H . We start with a review of the relevant literature before defining the functional sign and spatial ranks function.

### 3.1 The functional spatial rank

A spatial approach to multivariate and functional data appeared as early as 1983, when the spatial median was used for robust location estimation for two dimensional spatial data.^15^ The development of non-parametric geometrical approaches led to the introduction of multivariate spatial quantiles^16^ and the multivariate spatial depth function.^17^

The functional spatial depth (FSD), proposed by Chakraborty and Chaudhuri,^18^ extends the notion of spatial depth from *d*-dimensional multivariate space $R^{d}$ into infinite dimensional spaces. As a result, the multivariate spatial depth function, $SD\left(x\right)=1-\left\|E\left\{\left(x-X\right)/\left\|x-X\right\|\right\}\right\|$ , where $SD\left(x\right)$ is the spatial depth of $x\in R^{d}$ with respect to the probability distribution of a random vector $X\in R^{d}$ , can be extended naturally to any Hilbert space H . Thus, for any x∈H and a random element X∈H , the FSD(x) is defined based on same expression as *SD*, where $\left\|.\right\|$ is the usual norm in H and the expectation E is estimated based on the Bochner integral.^18,19^

The spatial depth function has been used to provide a nonparametric description of functional data, by using the functional version of spatial depth to identify some nonparametric descriptive features such as sample median and quantile curves.^20^

The functional spatial median has been of particular interest to investigators. For example, Cardot et al.^21^ used an averaged stochastic gradient algorithm to compute the functional spatial median in a Hilbert space in a fast way. And this functional spatial median has been used as a robust measure of center for a data set of electricity loading curves.^22^ The kernelized functional spatial depth (KFSD) has been proposed^23^ for the classification of functional data. It is based on the functional spatial depth introduced by Serfling and Wijesuriya.^17^ In addition, the functional K-nearest neighbour classifier has been used in this work as a benchmark procedure.

Suppose that X is a random variable with values in an infinite dimensional space or functional space. For instance, the stochastic process $X=\{X\;\left(t\right);t\in T\}$ ; where T⊂R is a good example for the functional variable X , which takes values in some Hilbert space H of functions defined on some set T , where T represents an interval of time, of wavelengths or any other subset of R .^1^ We now define the functional spatial rank. Suppose that $\left\{X_{1}\left(t\right),X_{2}\left(t\right),\ldots ,X_{n}\left(t\right)\right\}$ is a functional dataset based on the functional random variables $\left\{X_{1}\left(t\right),X_{2}\left(t\right),\ldots ,X_{n}(t)\right\}$ that take values in  H , and t is defined on some continuous interval T , then the population functional spatial rank function for the curve $x\left(t\right)\in H$ is defined as

(1) $${\mathrm{FSR}}_{F}\left(x\left(t\right)\right)=E\left[\frac{x\left(t\right)-X\left(t\right)}{\left\|x\left(t\right)-X\left(t\right)\right\|}\right]$$

where $\left\|x\left(t\right)\right\|$ is the $l_{2}$ norm

(2) $$\left\|x\left(t\right)\right\|={\left\{\int_{T}^{}{\left\{x(t)\right\}}^{2}dt\right\}}^{\frac{1}{2}}$$

for an infinite dimensional space.

In practice, the curves are observed at a finite set of points, so that there are discrete observations for each sample path $X_{i}\left(t\right)$ at a finite set of knots $\left\{t_{ij}:j=1,\ldots ,m_{i}\right\}$ . Thus we have $X_{i}\left(t\right)=\left\{X\left(t_{i1}\right),X\left(t_{i2}\right),\ldots X\left(t_{im_{i}}\right)\right\}$ , and for shorthand $X_{ij}=X\left(t_{ij}\right)$ . Here, we consider regularly sampled curves, where the evaluation points t∈T are fixed for each curve, with the same length and knots, so that $\left\{t_{ij}:j=1,\ldots ,m\right\}$ and i=1,…,n . The corresponding $l_{2}$ norm of ${\mathrm{FSR}}_{F_{n}}$ denoted by ${\mathrm{FSRN}}_{F_{n}}$ is given by $\left\|{\mathrm{FSR}}_{F_{n}}(x\left(t\right))\right\|$ . Thus, if $\left\{X_{1}\left(t\right),X_{2}\left(t\right),\ldots ,X_{n}\left(t\right)\right\}$ is the set of curves regularly sampled at a finite set of observations, then the sample functional spatial rank of x(t) with respect to $X_{1}\left(t\right),X_{2}\left(t\right),\ldots ,X_{n}\left(t\right)$ is given by

(3) $${\mathrm{FSR}}_{F_{n}}\left(x\left(t\right)\right)=\frac{1}{n}\sum_{i=1}^{n}\frac{x\left(t\right)-X_{i}\left(t\right)}{\left\|x\left(t\right)-X_{i}\left(t\right)\right\|}$$

As a vector, the functional spatial rank provides information on the centrality of an observed curve and its direction. The $l_{2}$ norm $\left\|{\mathrm{FSR}}_{F_{n}}(x\left(t\right))\right\|$ , which is bounded to lie in the interval [0, 1), provides a measure of “distance” of ${\mathrm{FSR}}_{F_{n}}(x\left(t\right))$ from the spatial median of the functional data. Thus, when the $\left\|{\mathrm{FSR}}_{F_{n}}(x\left(t\right))\right\|$ is close to zero, the $x\left(t\right)$ will be close to the spatial median. In contrast, if $\left\|{\mathrm{FSR}}_{F_{n}}(x\left(t\right))\right\|$ is close to one, the $x\left(t\right)$ could be an outlier curve and potentially provides the basis for an approach to outlier detection.

Clearly it requires deciding upon a suitable cut-off for an outlier and one approach is to trim the sample of a proportion of curves with the highest ${\mathrm{FSRN}}_{F_{i}}\left(x\left(t\right)\right)$ . Thus trimming the sample of the top 1%, 2%, 5%, and 10%, has been used to investigate the stability of the clusters when a parametric model-based clustering approach has been used.^24,25^

A simpler approach is to derive the cut-off, C based on the upper whisker of the boxplot of ${\mathrm{FSRN}}_{F_{i}}\left(x\left(t\right)\right)$ using the formula $C=Q_{3}+(1.5\times \mathrm{IQR})$ where $Q_{3}$ is the upper quartile and IQR is the interquartile range ( $Q_{3}-Q_{1}$ ) when $Q_{1}$ is the lower quartile. Those curves with ${\mathrm{FSRN}}_{F_{i}}\left(x\left(t\right)\right)$ exceeding C are then considered outliers and this is the approach used here.

In principle, the functional spatial ranks can be applied for both regularly and irregularly sampled curves, where the functional spatial ranks are supposed to be calculated in general concept using the integrations instead of the summations quantities, and then with a formal procedure and methods we can estimate the integral functions and get the estimated values of the functional spatial ranks. Alternatively, we may use some smoothing functions or spline coefficients to get an equal length of the irregularly sampled curves, and then we can use the above equations to obtain the functional spatial ranks of the irregularly sampled curves.

### 3.2 Functional spatial ranks classifier

Before introducing the forward search algorithm, we consider the problem of classifying functional data to particular clusters. In general, it is important to assess whether the curves have been appropriately assigned to a cluster and whether they remain unassigned to any cluster. A further problem that may arise with some algorithms classifying functional data is when some curves are assigned to more than one cluster.

Clearly it is desirable to have a mechanism of assigning each curve in the functional data to an appropriate cluster. Here we use a nonparametric classifier based on the functional spatial ranks that is applied after determining the number of clusters. Assuming we have *k* groups of observations, with population distributions *F*_1_, *F*_2_, … , *F*_*k*_, we may assign $x\left(t\right)$ to the group in which the $l_{2}$ norm of the functional spatial ranks based on *F_i_* is smallest such that

(4) $${\mathrm{FSRN}}_{F_{i}}\left(x\left(t\right)\right)=\underset{1\leq j\leq k}{\min}{\mathrm{FSRN}}_{F_{j}}\left(x\left(t\right)\right)$$

where i≠j,1≤i≤k . Thus, the forward search algorithm that follows identifies the number of clusters then applies the classifier in equation (4) to assign each curve to the most suitable cluster.

### 3.3 The forward search based on functional spatial rank algorithm

Let S(m) be a subset from the observed curves of size *m*. Define the functional spatial ranks of an individual curve corresponding to the subset S(m) as

(5) $$r_{i}\left(m\right)=\frac{1}{m}\sum_{j\in S(m)}^{m}\frac{X_{i}\left(t\right)-X_{j}\left(t\right)}{\left\|X_{i}\left(t\right)-X_{j}\left(t\right)\right\|}$$

where i=1,…,n . The functional forward search algorithm with functional spatial ranks (FSFSR) is as follows:

1. Selecting random starting points, the search is started with an initial subset S(m) with size *m* = 3.
2. Calculate the functional spatial ranks $r_{i}\left(m\right)$ of the curves in the subset S(m) .
3. Compute $r_{\min}(m)$ , where $r_{\min}\left(m\right)=\min∥r_{i}\left(m\right)∥;i\notin S\left(m\right),$ where $\left\|.\right\|$ is the Euclidean norm, such that $∥r_{i}\left(m\right)∥=\sqrt{{r_{i}\left(m\right)}_{1}^{2}+{r_{i}\left(m\right)}_{2}^{2}+\cdots +{r_{i}\left(m\right)}_{t}^{2}}$ .
4. Grow the subset S(m) to S(m+1) by taking m+1 curves $X_{i}\left(t\right)$ ’s, which correspond to the smallest m+1 norms $∥r_{i}(m)∥$ ’s, where $\left\|.\right\|$ is the Euclidean norm as defined in step 3. Set m=m+1 .
5. Repeat 2 − 4 until m=n−1 .
6. Plot $r_{\text{min}}(m)$ against the corresponding subset sizes, m to get the forward plot and identify the number of clusters.
7. Identify the subset size by finding the highest $r_{\text{min}}(m)$ around each peak and set m as the cluster size. To specify the membership of each group, we may stop the search at each peak and set the curves included in m as the cluster’s membership.
8. Apply the functional spatial ranks classifier in section 3.2 to confirm the assignment of each curve and allocate the unassigned/incorporated curves to the proper group.

Theoretically, the algorithm can be started with any number of random starting points, since it is not constrained with the rule of *m* = *d*+1 as in the multivariate case. However, using a small number of the random starting points improves the chances of detecting small clusters in the data; hence we start the search with *m* = 3 . The trade-off is that the search may produce a large number of peaks for small values of *m* and it may be difficult to determine number of clusters visually and their respective sizes.

The computation of *r_i_*(*m*) may be computationally expensive in very high dimensions and very large samples sizes. As a result, increasing the number of random start forward searches increases the computational time. In order to elucidate the structure of the data within a reasonable computational time, we use 100 randomly chosen initial subsets. However, for lower dimensions and small sample sizes, we may increase the number of random start forward searches to 200 or 300. This reduces the risk of missing a trajectory with a different pattern.

When the curves in S(m) belong to the same cluster, the $∥r_{i}(m)∥$ for a curve $X_{i}\left(t\right)$ in that cluster is expected to be smaller than that for a curve from a different cluster. Furthermore, as S(m) grows, we expect to see a jump in the magnitude of the rank function when the nearest point to S(m) is from a different cluster. So, we may determine the number of clusters and their sizes in the functional data using the forward plot based on the functional spatial ranks.

## 4 Numerical examples

In this section, we apply the FSFSR algorithm proposed in section 3.2 to some numerical examples. The first three examples are simulated data generated from three different models. The final two examples use data from real datasets.

To assess the performance of the FSFSR algorithm, it is important to recognize that the algorithm both identifies the number of clusters and assigns all the data to an appropriate cluster. Thus, any performance metric must capture both of these elements and penalize the performance when either it identifies an incorrect number of clusters or wrongly assigns data to a cluster. Thus, we use the following misclassification rate, which is similar to the classification error proposed by Meila.^26^

For *n* data points, suppose there are *r* true classes *T =* {*T_1_, T_2_, …, T_r_*}, and *k* clusters based on the clustering algorithm *C =* {*C_1_, C_2_, …, C_k_*}. And define the two vectors *A* and *B* such that *A* = {1, 2, … , k} and *B* = {1, 2, … , r}. Then the misclassification rate, H can be defined as

(6) $$H=1-\left(\frac{1}{n}\right)\max\left(\sum_{\left(i,j\right)\in A\times B}\left|C_{i}\cap T_{j}\right|\right)$$

with the condition that if the two terms $\left|C_{i}\cap T_{j}\right|$ and $\left|C_{t}\cap T_{u}\right|$ appear in the sum then *i = t* if and only if *j = u*. This guarantees the rows and columns of the matrix *A × B* contribute at most one element to the summation. Consequently, the term $\left|C_{i}\cap T_{u}\right|$ is set to zero if the term $\left|C_{i}\cap T_{j}\right|$ is one of the terms that maximizes the sum in parentheses. For *k *=* *1, the sum would contain only one term.

The adjusted Rand Index (ARI) is also popular metric used for measuring the performance of clustering algorithms and it has been included here for completeness.^27^ In contrast to H, which compares clusters by matching sets, the ARI compares clusters by counting the pairs of points in which the clusters agree or disagree. It also corrects for the expected value of the unadjusted Rand Index, where the expected value is based on a random choice of entries in the contingency table when the column and row totals are fixed.

For comparison, we considered six other methods for identifying the number of clusters and cluster sizes. Note in what follows the term in brackets, after the method name, corresponds to the function and package in **R**. The first method is model-based clustering (*mclust*)^28^ based on a Gaussian mixture model (GMM).^29^ The number of clusters is determined by the model which returns the largest Bayesian information criterion (BIC). For the second method, the *K-means*,^30^ the number of clusters needs to be set in advance, and here it corresponds to the number which returns the largest CH index.^31^ The third method is the high dimensional data clustering method (*HDDC*).^32^ It is a model-based clustering method also based on the GMM, where the number of clusters corresponds to the model which returns the largest BIC. The fourth method is the mixtures of probabilistic principle component analyzers (*MixtPPCA*)^33^ which again uses the model with the largest BIC to determine the number of clusters. The partitioning around medoids (*PAM*)^34^ is the fifth method considered. Here the number of clusters is selected based on the optimum average silhouette width.^35^ Finally, the sixth method is the functional high-dimensional data clustering method (*FunHDDC*) which is an adaptive method that uses the functional data directly and chooses the number of clusters based on the largest BIC value.^36^

The first five methods were implemented as a raw-data method with discretized data and as a filtering method based on 10 spline coefficients. All six methods were applied to the three simulated and two real datasets and compared with the FSFSR algorithm using the number of clusters identified, H^26^ and ARI.^27^

For the *K-means*^30^ and *HDDC*^32^ methods the initial partitioning of the data points is random, which may result in the values of the performance measures varying between different runs of the algorithm. For these cases, the algorithms were repeated 1000 times and the average over the repetitions has been calculated.

### 4.1 Simulated data examples

The first simulated data (model 1) consists of two groups. The first group includes curves that are generated from the process

(7) $$X\left(t\right)=m_{0}\left(t\right)+e\left(t\right)$$

with mean function $m_{0}\left(t\right)=-35(1-t)t^{1.4}$ and e(t) is a Gaussian process with mean 0 and $\mathrm{Cov}\left(X\left(s\right),X\left(t\right)\right)=0.3\exp\left(-\left|s-t\right|/0.4\right)$ . Here, t is a sequence of numbers between 0 and 1 with length 100. The second group consists of the generated curves from the process

(8) $$Y\left(t\right)=m_{1}\left(t\right)+e\left(t\right)$$

where the mean function $m_{1}\left(t\right)=-35t{\left(1-t\right)}^{1.4}$ , with both Gaussian process e(t) and $\mathrm{Cov}\left(X\left(s\right),X\left(t\right)\right)$ are defined as in $X\left(t\right)$ in equation (6). So, the mixture model $Z\left(t\right)$ consists of the two groups $X\left(t\right)$ and $Y\left(t\right)$ such that, $Z\left(t\right)=pY\left(t\right)+(1-p)X(t)$ , where p is the mixing proportion, which is the probability of an individual curve being generated by specific process.

**Figure 1.**
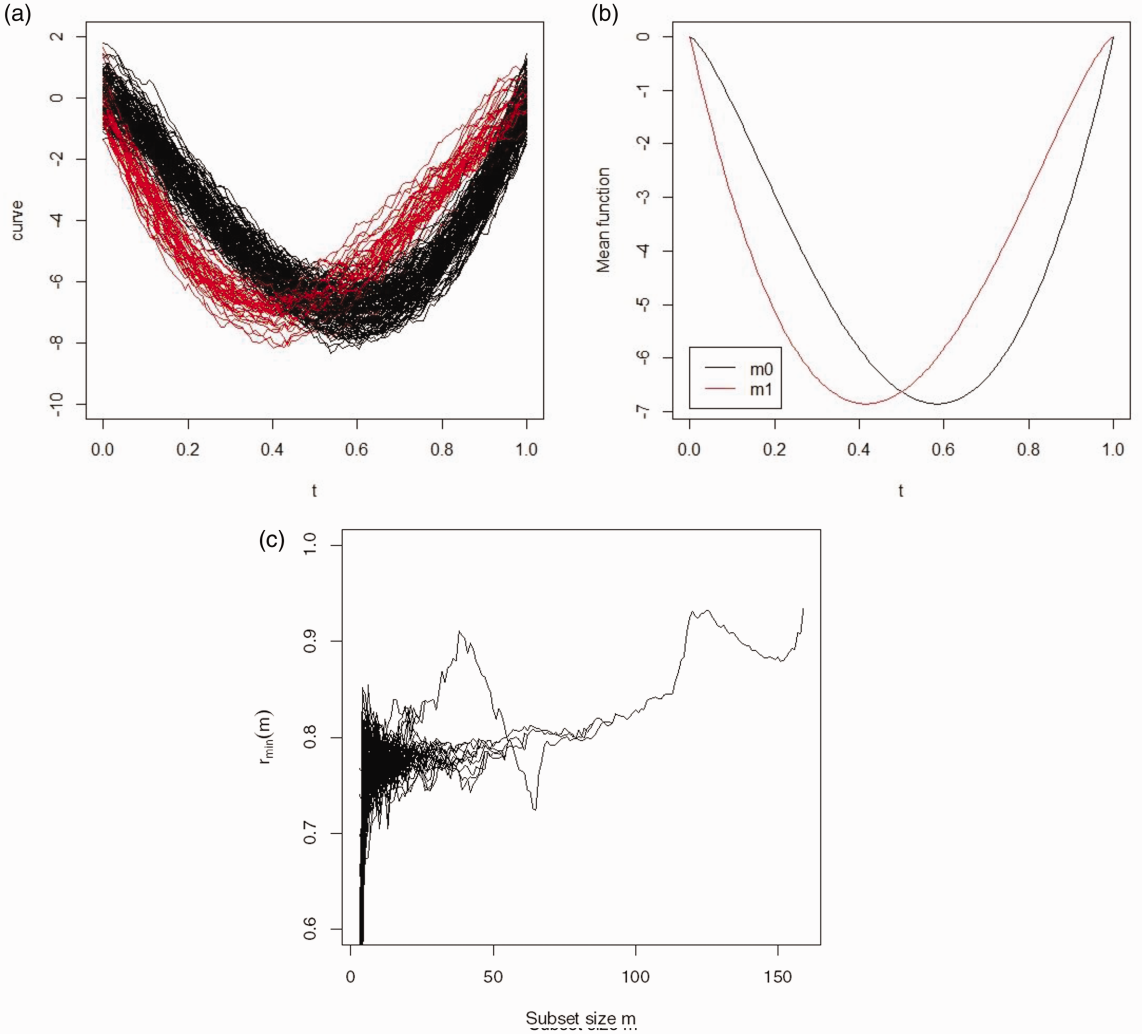
Simulated data, Model 1: (a) the observed curves with two groups, (b) the mean function, (c) the forward plot based on functional spatial ranks.

A mixing proportion of 0.25 was used to generate the two clusters with a sample size *n* =160 curves. The black curves, in Figures 1(a), represent the first group, and the red curves represent the second. In Figure 1(b), the mean function of model 1 is given. As the sample size is 160 and the mixing proportion is 0.25, the first cluster includes 40 curves and the second cluster includes 120 curves. Panel (c) of Figure 1 gives the forward plot of the simulated functional data based on the functional spatial ranks. It can be seen from panel (c), there are two maxima at sizes *m *=* *38 and 120, which suggests the data have been correctly divided into two groups. To identify the membership of the two clusters, we stopped the algorithm at *m *=* *38 and *m *=* *120. Before applying the classifier in step 7 of the algorithm, two curves remained unassigned. These were assigned to each cluster after applying the classifier resulting in two clusters of size 39 and 121, respectively. Comparing the clusters label with the simulated classes label, 159 curves out of 160 have been assigned correctly. This gives an H of 0.00625, and an ARI of 0.973.

In Table 1 the performances of all the algorithms are summarized. Many of the algorithms identified the correct number of clusters and achieved perfect classification. However, three algorithms (*mclust*, *HDDC* and MixtPPCA) when implemented as raw-data methods with discretized data returned an incorrect number of clusters. This affected their respective misclassification rates and adjusted Rand indices.

**Table 1.** Comparison of different clustering approaches applied to Model 1.

Clustering method	No. of clusters	Cluster sizes	H	ARI
FSFSR (a)	2	39, 121	0.00625	0.97331
GMM (mclust) “BIC” (a)	1	160	0.25000	0.00000
GMM (mclust) “BIC” (b)	2	40, 120	0.00000	1.00000
Kmeans based on CH index (a)^a^	2	40, 120	0.00000	1.00000
Kmeans based on CH index (b)^a^	2	40, 120	0.00000	1.00000
HDDC “BIC” (a)^a^	4	10, 26, 38, 86	0.24141	0.53795
HDDC “BIC” (b)^a^	2	40, 120	0.00019	0.99973
MixtPPCA “BIC” (a)	1	160	0.25000	0.00000
MixtPPCA “BIC” (b)	2	40, 120	0.00000	1.00000
PAM “Silhouette width” (a)	2	40, 120	0.00000	1.00000
PAM “Silhouette width” (b)	2	40, 120	0.00000	1.00000
FunHDDC “BIC”	2	39, 121	0.00625	0.97331

Note: When a method is followed by letter in parentheses it denotes the following: (a) = raw-data methods with discretized data; (b) = filtering methods using spline coefficients from10 splines.

^a^Results are based on the mean of 1000 repetitions.

In the second model, there are also two groups. The first group consists of curves generated from the process similar to equation (7) but with a different mean function: $m_{0}\left(t\right)=-35\left(1-t\right)t^{3}+4\left|\sin(25\pi t)\right|$ and e(t) is the same Gaussian process as defined in process (7). The second group is a smoothing of the curves of the first group, and it consists of spline approximations (with eight knots) of the trajectories in the first group. This in fact makes it more difficult to discriminate the overlap between the two clusters. Similarly, we set *n* =160 and the mixing proportion to 0.25.

**Figure 2.**
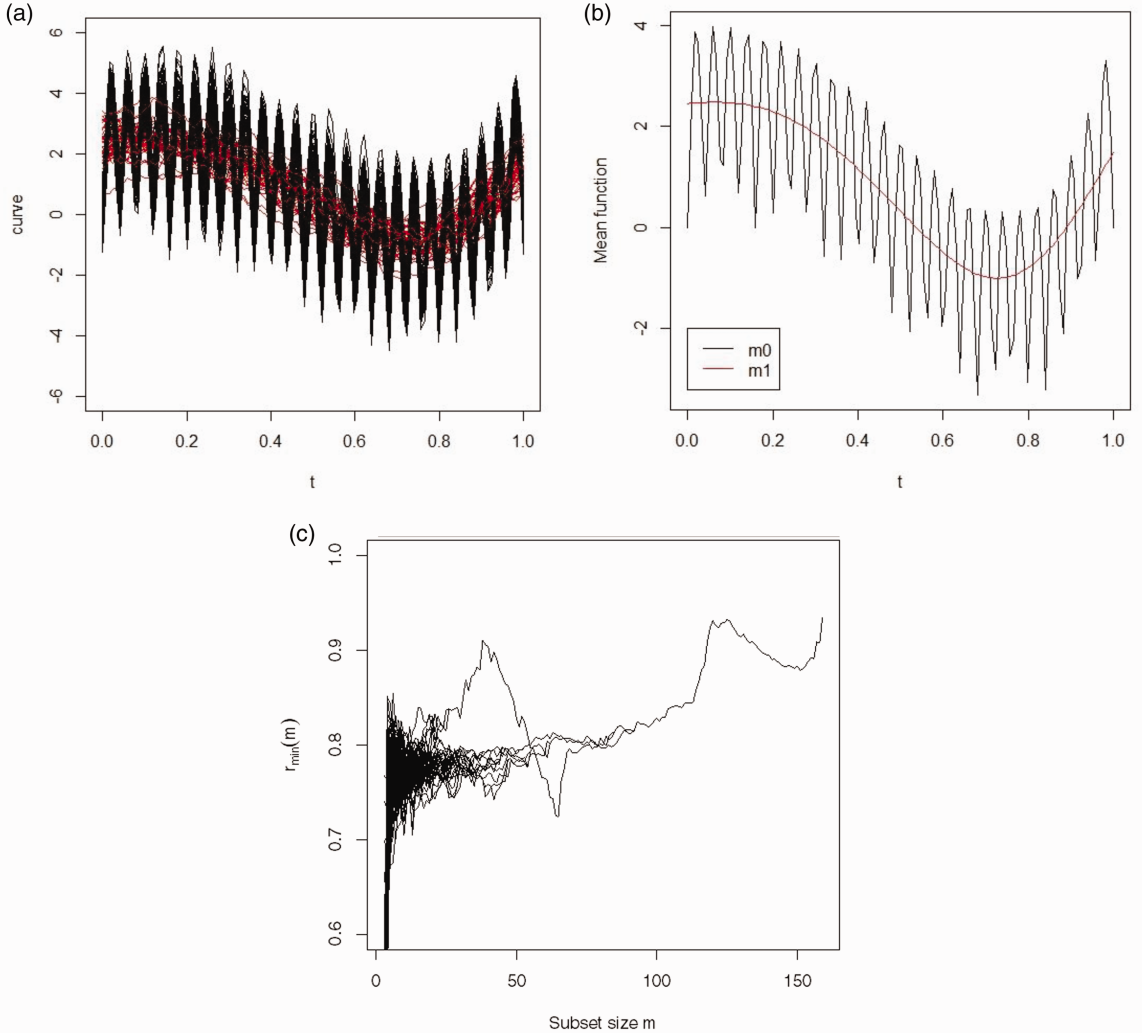
Simulated data, Model 2: (a) the observed curves with two groups, (b) the mean function, (c) the forward plot based on functional spatial ranks.

Figure 2(a) shows the simulated curves from the first cluster (black) and second cluster (red). The corresponding mean functions are shown in Figure 2(b). The forward plot based on the functional spatial ranks is shown in Figure 2(c). Again we can clearly see two peaks around *m *=* *40 and 117, and three curves (81, 120 and 148) have not been assigned to a cluster before the classification step. Applying the classifier assigns all three curves to *S*(*m = *117), thus resulting in two clusters of sizes 40 and 120. Furthermore, all 160 curves have been classified correctly so H is 0 and the ARI is 1. In Table 2 it can be seen that including FSFSR 7/12 algorithms identified the correct number of clusters. In 3/7 which identified the correct number of clusters, H was above 0.31 and the ARIs were between 0.02 and 0.04.

**Table 2.** Comparison of different clustering approaches applied to Model 2.

Clustering method	No. of clusters	Cluster sizes	H	ARI
FSFSR (a)	2	40, 120	0.00000	1.00000
GMM (mclust) “BIC” (a)	2	40, 120	0.00000	1.00000
GMM (mclust) “BIC” (b)	1	160	0.25000	0.00000
Kmeans based on CH index (a)^a^	2	40, 120	0.00000	1.00000
Kmeans based on CH index (b)^a^	2	79, 81	0.39443	0.04004
HDDC “BIC” (a)^a^	5	9, 19, 30, 40, 62	0.29649	0.43703
HDDC “BIC” (b)^a^	1	160	0.25000	0.00000
MixtPPCA “BIC” (a)	1	160	0.25000	0.00000
MixtPPCA “BIC” (b)	1	160	0.25000	0.00000
PAM “Silhouette width” (a)	2	40, 120	0.00000	1.00000
PAM “Silhouette width” (b)	2	63, 97	0.40625	0.01967
FunHDDC “BIC”	2	29, 131	0.31875	0.03015

Note: When a method is followed by letter in parentheses it denotes the following: (a) = raw-data methods with discretized data; (b) = filtering methods using spline coefficients from10 splines.

^a^Results are based on the mean of 1000 repetitions.

For the third model, we combine the two previous models so there are three clusters. The first cluster consists of the generated curves from the process defined in equation (7) but with a different mean function: $m_{0}\left(t\right)=-35\left(1-t\right)t^{1.4}+4\left|\sin(25\pi t)\right|$ , with both the Gaussian process e(t) and $\mathrm{Cov}\left(X\left(s\right),X\left(t\right)\right)$ defined as in $X\left(t\right)$ in equation (6). The second cluster is a smoothing of the curves of the first cluster, and it is made of spline approximations (with eight knots) of the trajectories in the first cluster. The third cluster is derived from equation (8) in model 1.

**Figure 3.**
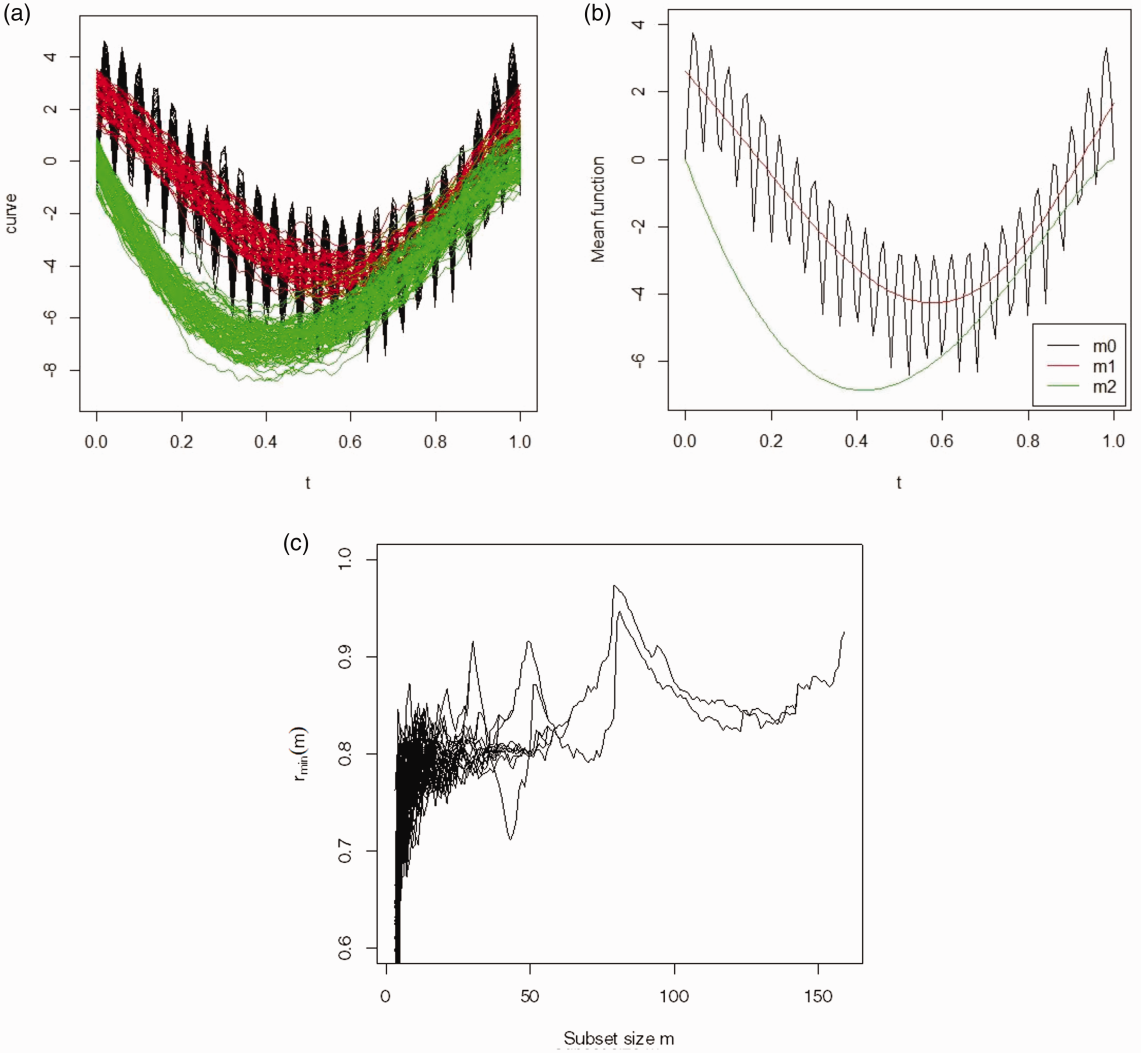
Simulated data, Model 3: (a) the observed curves with three groups, (b) the mean function, (c) the forward plot based on functional spatial ranks.

The three simulated clusters have sizes 30, 50 and 80. Figure 3(a) and (b) shows the respective simulated curves for the clusters and their mean functions. In Figure 3(c), the forward plot again demonstrates that the functional forward search algorithm has identified the correct number of clusters. There are three peaks at *m *=* *30, 49 and 79. In addition, there are two unassigned curves and the classifier in step 7 assigns both of them to cluster *S(m = *49*)* giving three clusters of sizes to 30, 51 and 79. Out of 160 curves in the sample, 159 curves have been assigned correctly, resulting in an H of 0.00625 and ARI of 0.978.

A comparison with the other algorithms is provided in Table 3. It can be seen that the FSFSR algorithm is the only algorithm which identifies the correct number of clusters with near perfect classification. In contrast, the other algorithms suffer significant misclassification rates although the adjusted Rand indices are less affected with many returning a value of 0.764.

**Table 3.** Comparison of different clustering approaches applied to Model 3.

Clustering method	No. of clusters	Cluster sizes	H	ARI
FSFSR (a)	3	30, 51, 79	0.00625	0.97843
GMM (mclust) “BIC” (a)	1	160	0.50000	0.00000
GMM (mclust) “BIC” (b)	2	80, 80	0.68750	0.76379
Kmeans based on CH index (a)^a^	2	80, 80	0.68750	0.76379
Kmeans based on CH index (b)^a^	2	80, 80	0.68750	0.76379
HDDC “BIC” (a)^a^	8	7, 12, 13, 14, 16, 22, 28, 48	0.67942	0.54742
HDDC “BIC” (b)^a^	2	80, 80	0.68750	0.76379
MixtPPCA “BIC” (a)	1	160	0.50000	0.00000
MixtPPCA “BIC” (b)	2	80, 80	0.68750	0.76379
PAM “Silhouette width” (a)	2	80, 80	0.68750	0.76379
PAM “Silhouette width” (b)	2	80, 80	0.68750	0.76379
FunHDDC “BIC”	2	80, 80	0.68750	0.76379

Note: When a method is followed by letter in parentheses it denotes the following: (a) = raw-data methods with discretized data; (b) = filtering methods using spline coefficients from10 splines.

^a^Results are based on the mean of 1000 repetitions.

### 4.2 Real data examples

In this section we apply the FSFSR algorithm to two real datasets. The first dataset is known as the ECG data and is taken from the UCR Time Series Classification and Clustering Archive.^37^ The dataset consists of 200 electrocardiograms from two groups of patients sampled at 96 time points, in which 133 are classified as normal and 67 as abnormal. The data consist of the ECG signals recorded between two electrodes during one heartbeat. The abnormal ECGs reflect a cardiac pathology known as a supraventricular premature beat.

The second dataset, known as the ‘DistalPhalanxOutlineCorrect’ data (hereon referred to as the Distal data) is also taken from the UCR Time Series Classification and Clustering Archive.^38,39^ It is designed to test the efficacy of hand and bone outline detection by an image processing algorithm. The outlines of the three bones of the middle finger in each image are summarized by a univariate series of 80 data points representing Euclidean distances of different points around the outline from a central point. Here, we consider the test sample of 276 images. There are two classes based on whether the bones have been correctly delineated by the image processing algorithm (115) or not (161) as determined by human evaluation.

**Figure 4.**
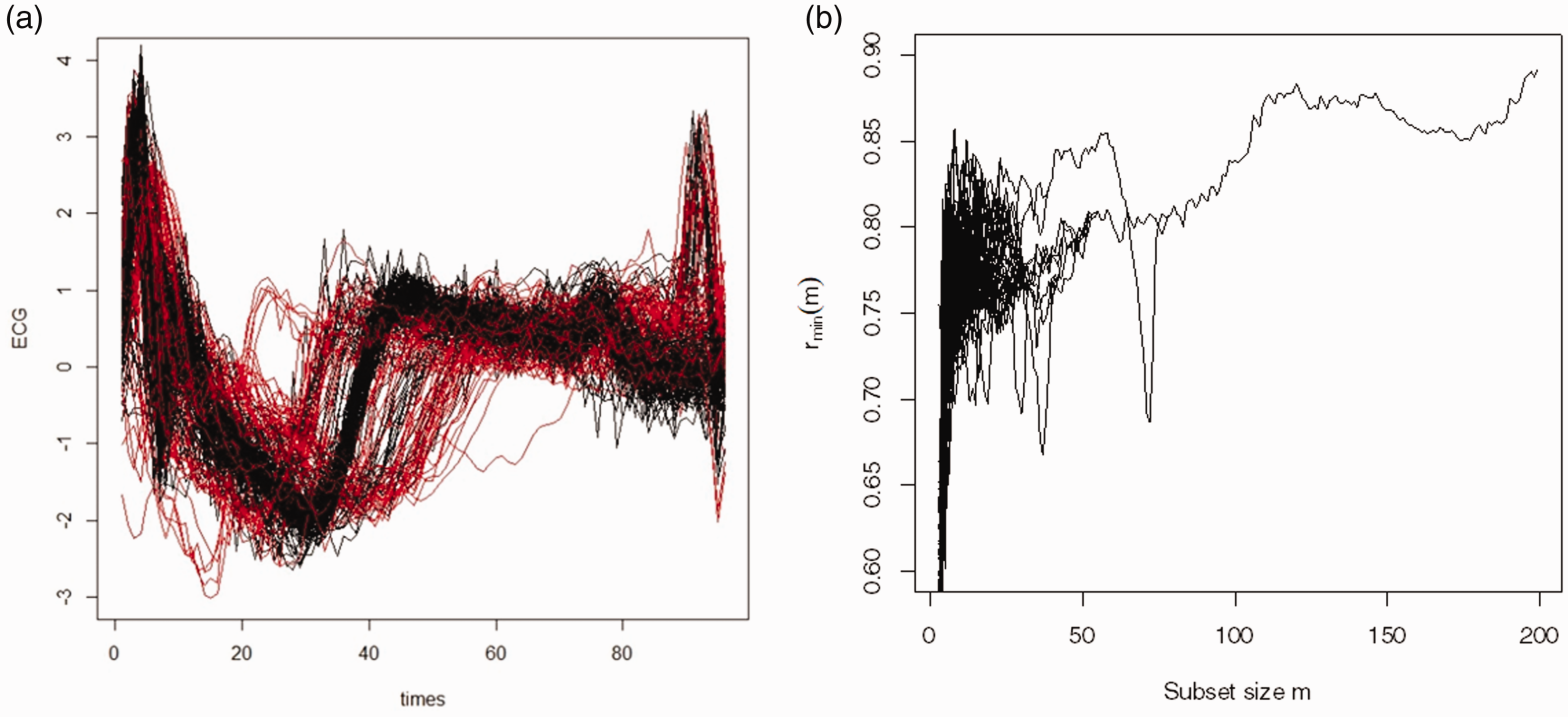
ECG data: panel (a) is the observed curves with two groups and panel (b) is the forward plot based on the functional spatial ranks. Two clusters are evident at subsets with sizes 58 and 120.

Figure 4(a) shows the observed curves for the ECG data, and the forward plot based on functional spatial rank. From Figure 4(b), two clusters are evident with peaks at 58 and the other at 120. This suggests that some of the observations have not been captured by either cluster. In order to identify the membership of each cluster, the forward search was stopped at the first peak (*m* = 58) to identify the subset *S*(*m* = 58). Similarly, stopping the search at *m* = 120 identifies the second cluster *S*(*m* = 120). Before applying the classifier in step 7 of the algorithm, 15 curves have been incorporated in both clusters and 37 curves have not been assigned to any cluster. Applying the functional spatial ranks classifier to these 52 curves classifies each curve to a unique cluster. As a result, H is 0.235 and the ARI is 0.264.

Table 4 gives the results for all the methods applied to the ECG data. It is clear that only the FSFSR algorithm gives the correct number of clusters (2) and has the lowest H (23.5%). Despite identifying an incorrect number of clusters, for many of the other methods the ARI is more favourable than the FSFSR algorithm. None is above 0.39 and this would suggest poor classification by all the algorithms; however, interpretation of the ARI is not straightforward as the baseline expected value for the Rand index varies as the contingency table varies.^40,41^

**Table 4.** Comparison of different clustering approaches applied to the ECG dataset.

Clustering method	No. of clusters	Cluster sizes	H	ARI
FSFSR (a)	2	58, 142	0.23500	0.26405
GMM (mclust) “BIC” (a)	1	200	0.33500	0.00000
GMM (mclust) “BIC” (b)	3	97, 41, 62	0.31500	0.37860
Kmeans based on CH index (a)^a^	3	111, 35, 54	0.32489	0.31411
Kmeans based on CH index (b)^a^	3	117, 48, 35	0.32000	0.32874
HDDC “BIC” (a)^a^	3	36, 51, 113	0.36217	0.32400
HDDC “BIC” (b)^a^	5	58, 14, 20, 17, 91	0.39474	0.37243
MixtPPCA “BIC” (a)	3	104, 61, 35	0.30000	0.36854
MixtPPCA “BIC” (b)	4	97, 39, 26, 38	0.37000	0.38710
PAM “Silhouette width” (a)	3	109, 55, 36	0.32000	0.33304
PAM “Silhouette width” (b)	4	118, 47, 19, 16	0.32500	0.33866
FunHDDC “BIC”	1	200	0.33500	0.00000

Note: When a method is followed by letter in parentheses it denotes the following: (a) = raw-data methods with discretized data; (b) = filtering methods using spline coefficients from10 splines.

^a^Results are based on the mean of 1000 repetitions.

**Table 5.** Comparison of different clustering approaches applied to the Distal dataset.

Clustering method	No. of clusters	Cluster sizes	H	ARI
FSFSR (a)	2	211, 65	0.23551	0.00512
GMM (mclust) “BIC” (a)	1	276	0.41667	0.00000
GMM (mclust) “BIC” (b)	4	140, 14, 53, 69	0.49275	0.10492
Kmeans based on CH index (a)^a^	2	95, 181	0.38406	0.04816
Kmeans based on CH index (b)^a^	2	103, 173	0.35542	0.07892
HDDC “BIC” (a)^a^	5	40, 119, 28, 27, 62	0.53986	0.08396
HDDC “BIC” (b)^a^	5	147, 64, 16, 46, 3	0.50362	0.06951
MixtPPCA “BIC” (a)	6	69, 55, 32, 38, 6, 76	0.63406	0.07866
MixtPPCA “BIC” (b)	3	75, 174, 27	0.46014	0.02750
PAM “Silhouette width” (a)	2	173, 103	0.36957	0.06328
PAM “Silhouette width” (b)	2	180, 96	0.37319	0.05888
FunHDDC “BIC”	1	276	0.41667	0.00000

Note: When a method is followed by letter in parentheses it denotes the following: (a) = raw-data methods. with discretized data; (b) = filtering methods using spline coefficients from 10 splines.

^a^Results are based on the mean of 1000 repetitions.

**Figure 5.**
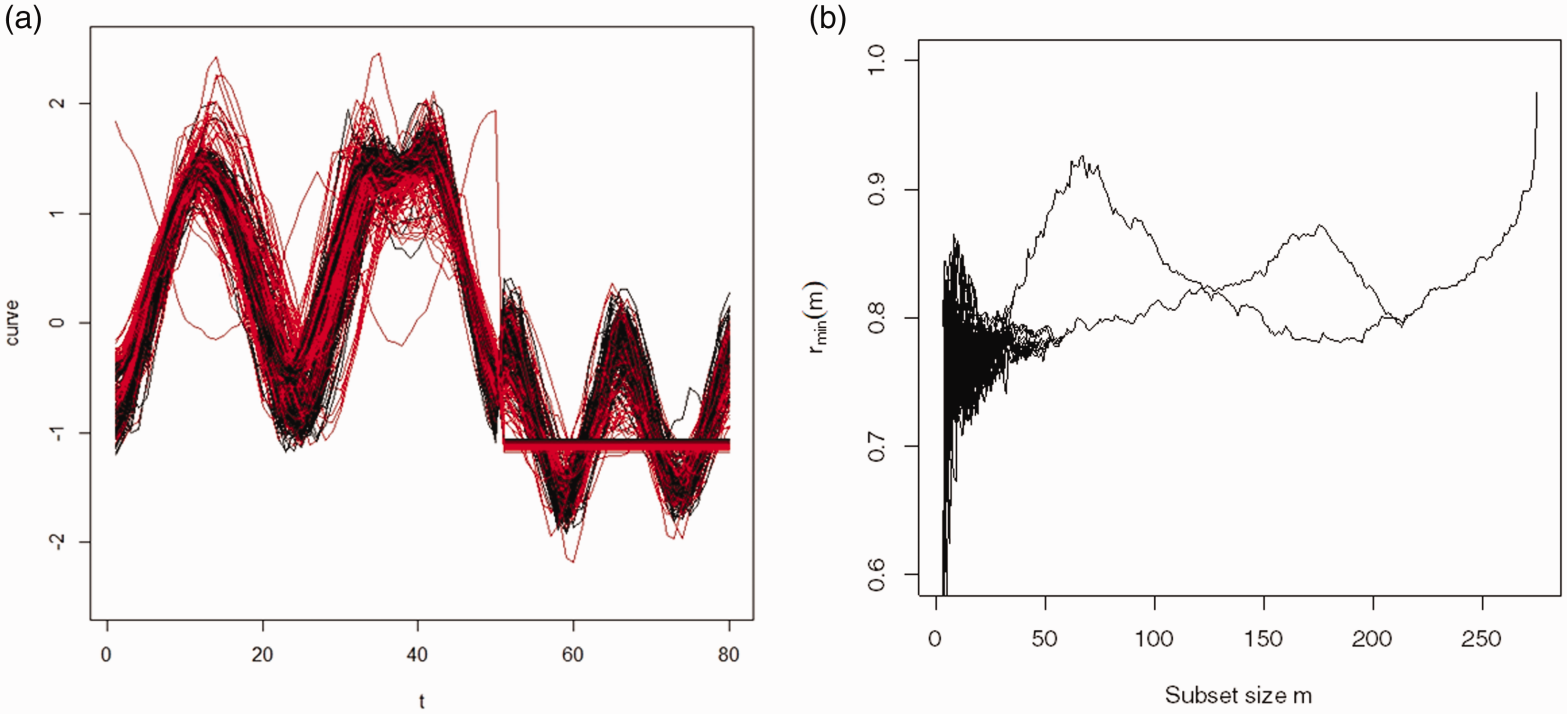
Distal data: panel (a) is the observed curves with two groups and panel (b) is the forward plot based on the functional spatial ranks. Two clusters are evident at subsets with sizes 67 and 175.

The Distal data curves for the 276 images are given in Figure 5(a). It is clearly seen that there is a high level of similarity between the two classes, which makes the distinction between them difficult. Figure 5(b) displays the forward plot based on the functional spatial rank for the Distal data. Two clusters are evident with two clear peaks at 67 and the other 175. Before applying the classifier in the algorithm, 36 curves remain unassigned to a cluster and two curves have been incorporated in both clusters. After step 7, each of these 38 curves has been assigned to a single appropriate cluster and H for the algorithm is 0.236.

It is clear from Figure 5(a) that one curve of the curves (number 220), which starts from the upper left and travels in a different direction from the other curves is a potential outlier. From the data, the cut-off C for outliers equals 0.9586 and for this curve, ${\mathrm{FSRN}}_{F_{i}}\left(x_{220}\left(t\right)\right)$ = 0.9726. On this basis, curve 220 may be considered an outlier in the Distal dataset.

Table 5 gives the results for the Distal data, and as can be seen, only five methods including the FSFSR algorithm gave the correct number of clusters (2). The FSFSR algorithm again records the lowest H (23.6%). For the ARI, *mclust* method based on 10 spline coefficient has the highest ARI with 0.104 but identified four clusters.

## 5 Discussion

In this paper, we have proposed a new forward search algorithm for clustering functional data. It is an extension to the forward search methodology based on spatial ranks that has been introduced for the multivariate case.^12^ It may be used to identify the number of clusters in the underlying functional data and does not require any preprocessing of the data, nor the need to perform data registration or dimension reduction before clustering. Furthermore, it may be used in cases when the number of variables exceeds the number of observations or when the cluster size is less than the number of variables – this contrasts traditional forward searches based on Mahalanobis distances.

An important element of the algorithm is the inclusion of a classifier. This allows the classification of all curves to an appropriate cluster, even when in the early steps some have either not been assigned or have been assigned to more than one cluster.

As the FSFSR algorithm both identifies clusters and classifies functional data, any reasonable comparison should be with methods that are also capable of clustering and classifying. Equally, it is important that the metric used to gauge performance also adequately captures both clustering and classifying. To this end, we used the misclassification rate H, which penalizes methods that identify an incorrect number of clusters as well as assessing the error in classification and the adjusted Rand index (ARI) which is a popular metric used in classification and clustering.

For the simulated examples, the algorithm was able to identify correctly the number of clusters and the number of simulated curves in each cluster with an H of no more than 0.0063. Indeed for the third, more complex simulated example, it was the only algorithm to correctly identify the number of clusters with a near perfect H and ARI score.

For the two real examples, the FSFSR algorithm identified the correct number of clusters and had the lowest H amongst all the methods. However, in the last example it also had one of the poorest ARI scores and illustrates some of the shortcomings when using these metrics for comparing algorithms. It is clear from the real data examples that when an incorrect number of clusters are returned, H penalizes algorithms more severely than the ARI. In contrast, the ARI adjusts for correct classification by chance which should, in principle, give it an advantage over H.^26,40,41^ However, since the baseline expected Rand index may be different between two different partitions of the data, it is not clear if two algorithms were to return similar values for the ARI that this would represent equivalence in performance.^26,40,41^ Thus comparing performances can be difficult using this metric.

One of the limitations in the proposed algorithm is that, in order to identify the subset size correspondence to each peak in the trajectories of the random starts, we have to find the highest $r_{\min}(m)$ around each peak and set m as the cluster size. Currently, we stop the search at each peak, and then identify the subset size and its membership. However, there is the potential to automate this process using ideas contained in Cerioli et al.^42^ and this requires further research.

Several authors have demonstrated the use of the forward search based around a Mahalanobis distance metric to detect outliers on multivariate data.^43–47^ Distributional results are known for the Mahalanobis distance and the minimum Mahalanobis distance allowing inferential statements to be made. In particular, percentile envelopes that contain most of the data may be estimated so that outlier points lie outside the enveloped region. In contrast, the forward search proposed here has been developed in a nonparametric framework. This makes it more difficult to use envelopes from order statistics based on distributional assumptions and approximations for unscaled distances and asymptotic results and requires further research.

When there are a large number of clusters the proposed forward search may produce too many peaks and this may make it difficult to determine the number of clusters and their sizes. Furthermore, the selection of random starting points as used here can result in multiple peaks which makes it hard to identify small clusters; hence, a more effective divisive strategy can instead be used.^43–47^

Although spatial ranks are invariant under orthogonal transformations, they are not invariant under general affine transformations of the data, thus the proposed algorithm is not affine invariant. An affine invariant version of the algorithm could be formulated based on affine invariant spatial ranks^48^ and this could improve the results if the scales of the clusters were different for instance. However, this would make the algorithm computationally expensive and greatly increase the process time and as a result we did not use any affine invariant versions of spatial ranks here.

The treatment of outliers is important in cluster analysis as their presence may indicate the existence of clusters or populations not specified in the initial analysis. Equally they may arise due to errors in recording of some form. Potentially both can distort the process of cluster identification and data classification and, in the case of model-based approaches, bias the estimates of associated parameters. This has led some investigators to propose methods such as ‘trimming’ the data of outliers as part of the analysis.^24,25^

The first part of the FSFSR algorithm, the forward search, identifies the number of clusters and their constituents. In some cases, some of the data may remain unclassified at this stage of the algorithm, as in the case of the Distal dataset. These unclassified data tend to have functional spatial rank norms ( ${\mathrm{FSRN}}_{F_{i}}\left(.\right)$ ) that are larger than the classified data and further away from the spatial median. Although the FSFSR algorithm assigns these curves to an appropriate cluster, the implicit assumption is that the forward search has identified the correct number of clusters. Without further investigation, some of these curves could be unidentified outliers and indicate, potentially, the existence of other clusters.

Here we used the calculated upper whisker of the box plot distribution as the threshold for outliers and this identified one potential outlier in the Distal dataset. Although the source of the outlier is unclear, in itself it would be insufficient to conclude that it arose from another population. Other approaches to the detection and treatment of outliers have been described and this remains an active area of research.^24,25,43,49^

In this study, both simulated and real datasets were used to compare the proposed algorithm with existing methods. One drawback when dealing with real data is that the identification of ‘true clusters’ is often not as clearly defined as in simulated datasets. Thus, errors in the reference classes, and the intrinsic dependence of the reference classification on the problem at hand, may diminish the effectiveness of this approach as a benchmarking procedure.^50,51^

In this study, we have proposed the FSFSR algorithm and demonstrated its potential as a clustering and classifying method for functional data. A more extensive evaluation of its performance across a greater range of examples is clearly necessary. However, as a data-driven non-parametric method, the approach proposed here is free from assumptions on the underlying distributions of the data and we believe it represents a significant development in functional data analysis.
